# Crystal structure of 2-phenyl­ethyl­amin­ium 4-nitro­phenolate monohydrate

**DOI:** 10.1107/S1600536814025318

**Published:** 2014-11-21

**Authors:** N. Swarna Sowmya, S. Sampathkrishnan, S. Sudhahar, R. Mohan Kumar, G. Chakkaravarthi

**Affiliations:** a Department of Applied Physics, Sri Venkateswara College of Engineering, Chennai 602 117, India; b Department of Physics, Presidency College, Chennai 600 005, India; c Department of Physics, CPCL Polytechnic College, Chennai 600 068, India

**Keywords:** crystal structure, 2-phenyl­ethyl­aminium, 4-nitro­phenolate, hydrated salt, O—H⋯O and N—H⋯O hydrogen bonds, π–π stacking inter­actions

## Abstract

In the title hydrated mol­ecular salt, C_8_H_12_N^+^·C_6_H_4_NO_3_^−^·H_2_O, the conformation of the side chain in the cation is *anti* [C—C—C—N = 179.62 (12)°] and the dihedral angle between the aromatic ring and the nitro group in the anion is 3.34 (11)°. In the crystal, the components are linked by O—H⋯O and N—H⋯O hydrogen bonds, generating (10-1) sheets, which feature *R*_4_^4^(21) loops. The sheets inter­act by weak aromatic π–π stacking inter­actions [centroid–centroid distance = 3.896 (3) Å], forming a three-dimensional network.

## Related literature   

For related structures, see: Kanagathara *et al.* (2012 ▶); Lejon *et al.* (2006 ▶); Sankar *et al.* (2014 ▶); Smith *et al.* (2003 ▶).
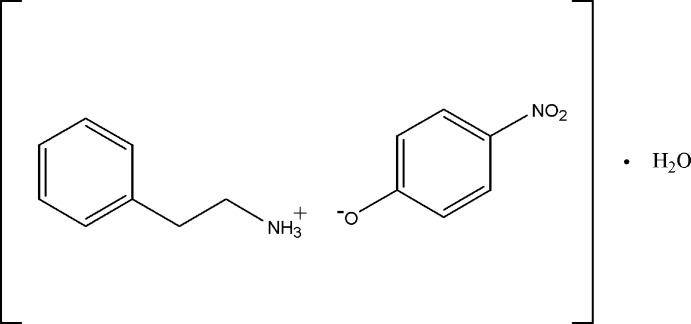


## Experimental   

### Crystal data  

C_8_H_12_N^+^·C_6_H_4_NO_3_^−^·H_2_O
*M* *_r_* = 278.30
Monoclinic, 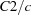

*a* = 30.381 (5) Å
*b* = 6.100 (4) Å
*c* = 21.357 (5) Å
β = 131.876 (5)°
*V* = 2947 (2) Å^3^
*Z* = 8
Mo *K*α radiation
μ = 0.09 mm^−1^
*T* = 295 K
0.26 × 0.24 × 0.20 mm

### Data collection  

Bruker Kappa APEXII CCD diffractometer
Absorption correction: multi-scan (*SADABS*; Sheldrick, 1996 ▶) *T*_min_ = 0.976, *T*_max_ = 0.982
14174 measured reflections
3675 independent reflections
2748 reflections with *I* > 2σ(*I*)
*R*_int_ = 0.021

### Refinement  

*R*[*F* ^2^ > 2σ(*F* ^2^)] = 0.043
*wR*(*F* ^2^) = 0.124
*S* = 1.03
3675 reflections
200 parameters
6 restraints
H atoms treated by a mixture of independent and constrained refinement
Δρ_max_ = 0.21 e Å^−3^
Δρ_min_ = −0.19 e Å^−3^

### 

Data collection: *APEX2* (Bruker, 2004 ▶); cell refinement: *SAINT* (Bruker, 2004 ▶); data reduction: *SAINT*; program(s) used to solve structure: *SHELXS97* (Sheldrick, 2008 ▶); program(s) used to refine structure: *SHELXL97* (Sheldrick, 2008 ▶); molecular graphics: *PLATON* (Spek, 2009 ▶); software used to prepare material for publication: *SHELXL97*.

## Supplementary Material

Crystal structure: contains datablock(s) global, I. DOI: 10.1107/S1600536814025318/hb7318sup1.cif

Structure factors: contains datablock(s) I. DOI: 10.1107/S1600536814025318/hb7318Isup2.hkl

Supporting information file. DOI: 10.1107/S1600536814025318/hb7318Isup3.cml

**Fig. 1.**
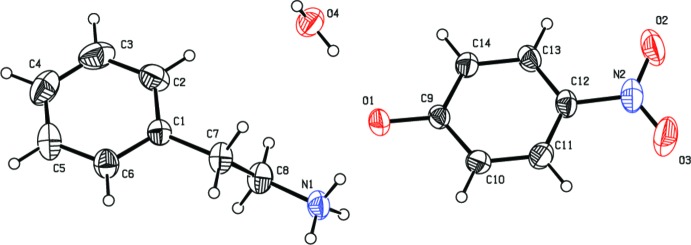
The molecular structure of (I), with 30% probability displacement ellipsoids for non-H atoms.

**Fig. 2.**
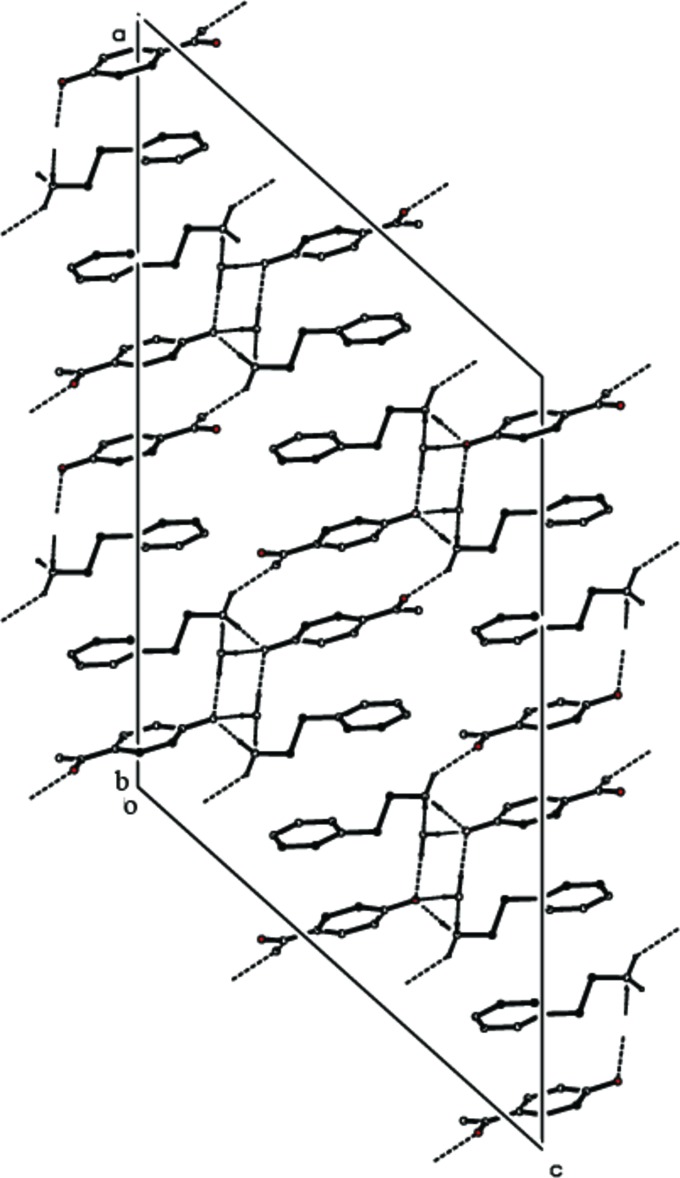
The packing of (I), viewed down b axis. Intermolecular Hydrogen bonds are shown as dashed lines. H atoms not involved in hydrogen bonding have been omitted.

. DOI: 10.1107/S1600536814025318/hb7318fig1.tif
The mol­ecular structure of (I), with 30% probability displacement ellipsoids for non-H atoms.

b . DOI: 10.1107/S1600536814025318/hb7318fig2.tif
The packing of (I), viewed down *b* axis. Inter­molecular Hydrogen bonds are shown as dashed lines. H atoms not involved in hydrogen bonding have been omitted.

CCDC reference: 1034880

Additional supporting information:  crystallographic information; 3D view; checkCIF report

## Figures and Tables

**Table 1:** Hydrogen-bond geometry (, )

*D*H*A*	*D*H	H*A*	*D* *A*	*D*H*A*
N1H1*A*O1	0.90(1)	1.81(1)	2.7108(17)	176(18)
O4H4*B*O1	0.84(1)	1.90(1)	2.7262(18)	173(2)
N1H1*B*O2^i^	0.90(1)	2.11(1)	2.8937(17)	145(15)
N1H1*C*O4^ii^	0.91(1)	1.84(1)	2.742(2)	172(18)
O4H4*A*O1^iii^	0.83(1)	1.93(1)	2.7574(16)	175(2)

Symmetry codes: (i) 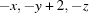; (ii) 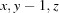; (iii) 
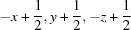
.

## supplementary crystallographic information

### S1. Structural commentary

The geometric parameters of the title compound (I) (Fig.1) are comparable with the reported similar structures (Kanagathara *et al.*, 2012; Sankar *et al.*, 2014; Lejon *et al.*, 2006; Smith *et al.*, 2003). The cation is protonated at N1 atom. The dihedral angle between the two benzene rings (C1—C6) and (C9—C14) is 3.71 (11)°. In the anion, the nitro group (N2/O2/O3) is twisted at an angle of 3.34 (11)° with the benzene ring (C9—C14).

### S2. Supra­molecular features

In the molecular structure, weak N—H···O and O—H···O hydrogen bonds link the cation, anion and water molecule which generates S(6) graph set motif. In the crystal structure, N—H···O and O—H···O hydrogen bonds link the anions, cations and water molecules into sheets, parallel to ac plane and further theses sheets are linked by O—H···O hydrogen bonds along [0 1 0] (Table 2 & Fig. 2). The N—H···O hydrogen bonds generates R_4_^4^(21) graph-set motif (Fig. 2).

The crystal structure also features weak C—H···π (Table 2) and π–π [Cg2···Cg2^i^ distance = 3.896 (3)Å; (i) -x,2-y,-z; Cg2 is the centroid of the C9—C14 ring] inter­actions to form a three dimensional network.

### S3. Synthesis and crystallization

2-Phenyl­ethyl­amine (1.26 g) and 4-nitro­phenol (1.39 g) were disssolved in methanol and colourless blocks of the title compound were grown by slow evaporation.

### S4. Refinement

Crystal data, data collection and structure refinement details are summarized in Table 1. The C-bound H atoms were positioned geometrically and refined using riding model, with C—H = 0.93 and 0.97 Å for CH_aromatic_ and CH_2_, respectively with U_iso_(H) = 1.2Ueq(C). The H atoms bound to O and N atoms were found in a difference map and refined isotropically, with U_iso_(H) = 1.5Ueq(O) and distance restraints: O—H = 0.82 (1)Å and N—H = 0.88 (1)Å. The components of the anisotropic displacement parameters in the direction of the bond between C3 and C4 were restrained to be equal within an effective standard deviation of 0.001 using the DELU command in SHELXL97 (Sheldrick, 2008).

### Crystal data

**Table:**

C_8_H_12_N^+^·C_6_H_4_NO_3_^−^·H_2_O	*F*(000) = 1184
*M**_r_* = 278.30	*D*_x_ = 1.254 Mg m^−^^3^
Monoclinic, *C*2/*c*	Mo *K*α radiation, λ = 0.71073 Å
Hall symbol: -C 2yc	Cell parameters from 359 reflections
*a* = 30.381 (5) Å	θ = 1.8–28.4°
*b* = 6.100 (4) Å	µ = 0.09 mm^−^^1^
*c* = 21.357 (5) Å	*T* = 295 K
β = 131.876 (5)°	Block, colourless
*V* = 2947 (2) Å^3^	0.26 × 0.24 × 0.20 mm
*Z* = 8	

### Data collection

**Table:**

Bruker Kappa APEXII CCD diffractometer	3675 independent reflections
Radiation source: fine-focus sealed tube	2748 reflections with *I* > 2σ(*I*)
Graphite monochromator	*R*_int_ = 0.021
ω and φ scan	θ_max_ = 28.4°, θ_min_ = 1.8°
Absorption correction: multi-scan (*SADABS*; Sheldrick, 1996)	*h* = −38→40
*T*_min_ = 0.976, *T*_max_ = 0.982	*k* = −7→8
14174 measured reflections	*l* = −28→28

### Refinement

**Table:**

Refinement on *F*^2^	Secondary atom site location: difference Fourier map
Least-squares matrix: full	Hydrogen site location: inferred from neighbouring sites
*R*[*F*^2^ > 2σ(*F*^2^)] = 0.043	H atoms treated by a mixture of independent and constrained refinement
*wR*(*F*^2^) = 0.124	*w* = 1/[σ^2^(*F*_o_^2^) + (0.0561*P*)^2^ + 0.954*P*] where *P* = (*F*_o_^2^ + 2*F*_c_^2^)/3
*S* = 1.03	(Δ/σ)_max_ < 0.001
3675 reflections	Δρ_max_ = 0.21 e Å^−^^3^
200 parameters	Δρ_min_ = −0.19 e Å^−^^3^
6 restraints	Extinction correction: *SHELXL97* (Sheldrick, 2008), Fc^*^=kFc[1+0.001xFc^2^λ^3^/sin(2θ)]^-1/4^
Primary atom site location: structure-invariant direct methods	Extinction coefficient: 0.0031 (5)

### Special details

**Table:**

Geometry. All esds (except the esd in the dihedral angle between two l.s. planes) are estimated using the full covariance matrix. The cell esds are taken into account individually in the estimation of esds in distances, angles and torsion angles; correlations between esds in cell parameters are only used when they are defined by crystal symmetry. An approximate (isotropic) treatment of cell esds is used for estimating esds involving l.s. planes.
Refinement. Refinement of F^2^ against ALL reflections. The weighted R-factor wR and goodness of fit S are based on F^2^, conventional R-factors R are based on F, with F set to zero for negative F^2^. The threshold expression of F^2^ > 2sigma(F^2^) is used only for calculating R-factors(gt) etc. and is not relevant to the choice of reflections for refinement. R-factors based on F^2^ are statistically about twice as large as those based on F, and R- factors based on ALL data will be even larger.

### Fractional atomic coordinates and isotropic or equivalent isotropic displacement parameters (Å^2^)

**Table:**

	*x*	*y*	*z*	*U*_iso_*/*U*_eq_	
C1	0.32367 (5)	0.9073 (2)	0.49756 (8)	0.0480 (3)	
C2	0.31910 (8)	1.1138 (3)	0.51903 (11)	0.0709 (4)	
H2	0.2885	1.2063	0.4777	0.085*	
C3	0.35989 (10)	1.1848 (3)	0.60213 (13)	0.0850 (5)	
H3	0.3564	1.3244	0.6159	0.102*	
C4	0.40509 (9)	1.0511 (4)	0.66371 (11)	0.0837 (5)	
H4	0.4322	1.0990	0.7192	0.100*	
C5	0.41005 (7)	0.8477 (4)	0.64310 (10)	0.0770 (5)	
H5	0.4407	0.7560	0.6846	0.092*	
C6	0.36972 (6)	0.7765 (3)	0.56068 (9)	0.0579 (3)	
H6	0.3738	0.6369	0.5476	0.070*	
C7	0.28038 (6)	0.8233 (3)	0.40816 (8)	0.0578 (3)	
H7A	0.2783	0.9273	0.3719	0.069*	
H7B	0.2949	0.6856	0.4052	0.069*	
C8	0.21941 (6)	0.7889 (3)	0.37618 (9)	0.0606 (4)	
H8A	0.2214	0.6890	0.4133	0.073*	
H8B	0.2039	0.9276	0.3763	0.073*	
C9	0.12653 (5)	1.0077 (2)	0.10944 (7)	0.0446 (3)	
C10	0.10095 (6)	0.8472 (2)	0.04638 (8)	0.0530 (3)	
H10	0.1191	0.7110	0.0601	0.064*	
C11	0.04981 (6)	0.8868 (2)	−0.03490 (8)	0.0567 (3)	
H11	0.0334	0.7782	−0.0756	0.068*	
C12	0.02310 (5)	1.0891 (2)	−0.05554 (7)	0.0498 (3)	
C13	0.04662 (5)	1.2508 (2)	0.00428 (8)	0.0521 (3)	
H13	0.0281	1.3867	−0.0105	0.062*	
C14	0.09722 (6)	1.2110 (2)	0.08547 (8)	0.0515 (3)	
H14	0.1126	1.3203	0.1257	0.062*	
N1	0.17925 (5)	0.6981 (2)	0.28992 (7)	0.0575 (3)	
H1A	0.1771 (8)	0.784 (3)	0.2537 (9)	0.081 (5)*	
H1B	0.1421 (5)	0.687 (3)	0.2687 (10)	0.079 (5)*	
H1C	0.1932 (8)	0.567 (2)	0.2897 (12)	0.087 (6)*	
N2	−0.03040 (5)	1.1357 (3)	−0.14047 (8)	0.0693 (4)	
O1	0.17576 (4)	0.97214 (16)	0.18676 (5)	0.0576 (3)	
O2	−0.05190 (5)	1.3215 (3)	−0.15745 (8)	0.0901 (4)	
O3	−0.05367 (6)	0.9919 (3)	−0.19366 (8)	0.1055 (5)	
O4	0.23008 (5)	1.3211 (2)	0.29355 (7)	0.0714 (3)	
H4A	0.2588 (7)	1.359 (4)	0.2990 (14)	0.107*	
H4B	0.2130 (9)	1.221 (3)	0.2576 (11)	0.107*	

### Atomic displacement parameters (Å^2^)

**Table:**

	*U*^11^	*U*^22^	*U*^33^	*U*^12^	*U*^13^	*U*^23^
C1	0.0467 (6)	0.0555 (7)	0.0463 (6)	−0.0004 (5)	0.0329 (6)	0.0024 (5)
C2	0.0845 (11)	0.0564 (9)	0.0748 (10)	0.0107 (8)	0.0544 (10)	0.0094 (7)
C3	0.1192 (15)	0.0617 (10)	0.0949 (12)	−0.0177 (8)	0.0801 (11)	−0.0224 (8)
C4	0.0833 (11)	0.1073 (15)	0.0598 (9)	−0.0306 (9)	0.0474 (9)	−0.0248 (8)
C5	0.0545 (8)	0.1111 (14)	0.0486 (8)	0.0072 (9)	0.0275 (7)	0.0073 (9)
C6	0.0506 (7)	0.0676 (9)	0.0533 (8)	0.0079 (6)	0.0337 (7)	0.0011 (6)
C7	0.0457 (7)	0.0815 (10)	0.0448 (7)	0.0011 (7)	0.0296 (6)	0.0002 (6)
C8	0.0491 (7)	0.0805 (10)	0.0542 (8)	0.0003 (7)	0.0353 (7)	−0.0014 (7)
C9	0.0342 (5)	0.0487 (7)	0.0426 (6)	−0.0014 (5)	0.0222 (5)	0.0067 (5)
C10	0.0499 (7)	0.0454 (7)	0.0563 (7)	0.0011 (5)	0.0324 (6)	0.0035 (6)
C11	0.0539 (7)	0.0599 (8)	0.0485 (7)	−0.0108 (6)	0.0310 (6)	−0.0067 (6)
C12	0.0355 (5)	0.0668 (8)	0.0396 (6)	−0.0029 (5)	0.0220 (5)	0.0074 (6)
C13	0.0403 (6)	0.0557 (7)	0.0517 (7)	0.0098 (5)	0.0273 (6)	0.0100 (6)
C14	0.0434 (6)	0.0512 (7)	0.0461 (7)	0.0006 (5)	0.0242 (6)	−0.0023 (5)
N1	0.0390 (6)	0.0707 (8)	0.0471 (6)	0.0026 (5)	0.0222 (5)	0.0091 (6)
N2	0.0460 (6)	0.1001 (11)	0.0440 (6)	−0.0063 (7)	0.0227 (6)	0.0111 (7)
O1	0.0395 (5)	0.0603 (6)	0.0456 (5)	0.0021 (4)	0.0170 (4)	0.0098 (4)
O2	0.0524 (6)	0.1087 (10)	0.0654 (7)	0.0168 (6)	0.0211 (6)	0.0326 (7)
O3	0.0852 (9)	0.1324 (13)	0.0444 (6)	−0.0151 (9)	0.0207 (6)	−0.0103 (7)
O4	0.0561 (6)	0.0691 (7)	0.0702 (7)	−0.0085 (5)	0.0344 (6)	−0.0119 (5)

### Geometric parameters (Å, º)

**Table:**

C1—C6	1.3761 (19)	C9—C10	1.4066 (19)
C1—C2	1.379 (2)	C9—C14	1.4084 (19)
C1—C7	1.5116 (18)	C10—C11	1.3729 (19)
C2—C3	1.392 (3)	C10—H10	0.9300
C2—H2	0.9300	C11—C12	1.378 (2)
C3—C4	1.367 (3)	C11—H11	0.9300
C3—H3	0.9300	C12—C13	1.377 (2)
C4—C5	1.358 (3)	C12—N2	1.4410 (17)
C4—H4	0.9300	C13—C14	1.3677 (18)
C5—C6	1.382 (2)	C13—H13	0.9300
C5—H5	0.9300	C14—H14	0.9300
C6—H6	0.9300	N1—H1A	0.901 (9)
C7—C8	1.5010 (19)	N1—H1B	0.895 (9)
C7—H7A	0.9700	N1—H1C	0.908 (9)
C7—H7B	0.9700	N2—O3	1.220 (2)
C8—N1	1.4795 (19)	N2—O2	1.235 (2)
C8—H8A	0.9700	O4—H4A	0.834 (9)
C8—H8B	0.9700	O4—H4B	0.836 (10)
C9—O1	1.3091 (14)		
			
C6—C1—C2	117.79 (14)	H8A—C8—H8B	108.0
C6—C1—C7	119.94 (13)	O1—C9—C10	121.89 (12)
C2—C1—C7	122.27 (13)	O1—C9—C14	121.15 (12)
C1—C2—C3	120.45 (16)	C10—C9—C14	116.96 (11)
C1—C2—H2	119.8	C11—C10—C9	121.59 (13)
C3—C2—H2	119.8	C11—C10—H10	119.2
C4—C3—C2	120.58 (17)	C9—C10—H10	119.2
C4—C3—H3	119.7	C10—C11—C12	119.33 (13)
C2—C3—H3	119.7	C10—C11—H11	120.3
C5—C4—C3	119.38 (16)	C12—C11—H11	120.3
C5—C4—H4	120.3	C13—C12—C11	120.97 (12)
C3—C4—H4	120.3	C13—C12—N2	118.50 (13)
C4—C5—C6	120.29 (17)	C11—C12—N2	120.53 (13)
C4—C5—H5	119.9	C14—C13—C12	119.79 (13)
C6—C5—H5	119.9	C14—C13—H13	120.1
C1—C6—C5	121.51 (15)	C12—C13—H13	120.1
C1—C6—H6	119.2	C13—C14—C9	121.35 (12)
C5—C6—H6	119.2	C13—C14—H14	119.3
C8—C7—C1	113.03 (10)	C9—C14—H14	119.3
C8—C7—H7A	109.0	C8—N1—H1A	112.1 (12)
C1—C7—H7A	109.0	C8—N1—H1B	111.8 (11)
C8—C7—H7B	109.0	H1A—N1—H1B	105.3 (16)
C1—C7—H7B	109.0	C8—N1—H1C	109.7 (12)
H7A—C7—H7B	107.8	H1A—N1—H1C	106.2 (16)
N1—C8—C7	111.13 (11)	H1B—N1—H1C	111.6 (17)
N1—C8—H8A	109.4	O3—N2—O2	121.46 (14)
C7—C8—H8A	109.4	O3—N2—C12	119.74 (16)
N1—C8—H8B	109.4	O2—N2—C12	118.79 (14)
C7—C8—H8B	109.4	H4A—O4—H4B	106 (2)
			
C6—C1—C2—C3	−0.3 (2)	C9—C10—C11—C12	0.6 (2)
C7—C1—C2—C3	179.97 (14)	C10—C11—C12—C13	−0.67 (19)
C1—C2—C3—C4	0.0 (3)	C10—C11—C12—N2	179.62 (12)
C2—C3—C4—C5	0.2 (3)	C11—C12—C13—C14	0.01 (19)
C3—C4—C5—C6	−0.1 (3)	N2—C12—C13—C14	179.73 (11)
C2—C1—C6—C5	0.4 (2)	C12—C13—C14—C9	0.8 (2)
C7—C1—C6—C5	−179.87 (13)	O1—C9—C14—C13	178.27 (12)
C4—C5—C6—C1	−0.2 (2)	C10—C9—C14—C13	−0.82 (19)
C6—C1—C7—C8	112.74 (15)	C13—C12—N2—O3	−176.42 (14)
C2—C1—C7—C8	−67.52 (18)	C11—C12—N2—O3	3.3 (2)
C1—C7—C8—N1	−177.62 (13)	C13—C12—N2—O2	3.03 (18)
O1—C9—C10—C11	−178.94 (12)	C11—C12—N2—O2	−177.25 (13)
C14—C9—C10—C11	0.15 (18)		

### Hydrogen-bond geometry (Å, º)

**Table:**

*D*—H···*A*	*D*—H	H···*A*	*D*···*A*	*D*—H···*A*
N1—H1*A*···O1	0.90 (1)	1.81 (1)	2.7108 (17)	176 (18)
O4—H4*B*···O1	0.84 (1)	1.90 (1)	2.7262 (18)	173 (2)
N1—H1*B*···O2^i^	0.90 (1)	2.11 (1)	2.8937 (17)	145 (15)
N1—H1*C*···O4^ii^	0.91 (1)	1.84 (1)	2.742 (2)	172 (18)
O4—H4*A*···O1^iii^	0.83 (1)	1.93 (1)	2.7574 (16)	175 (2)

Symmetry codes: (i) −*x*, −*y*+2, −*z*; (ii) *x*, *y*−1, *z*; (iii) −*x*+1/2, *y*+1/2, −*z*+1/2.
