# Supplementary figures and images for: Crystal structure of 2-phenyl­ethyl­amin­ium 4-nitro­phenolate monohydrate
Source: Acta Crystallogr Sect E Struct Rep Online. 2014 Nov 21;70(Pt 12):o1280. doi: 10.1107/S1600536814025318 (PMC4257380; doi:10.1107/S1600536814025318)

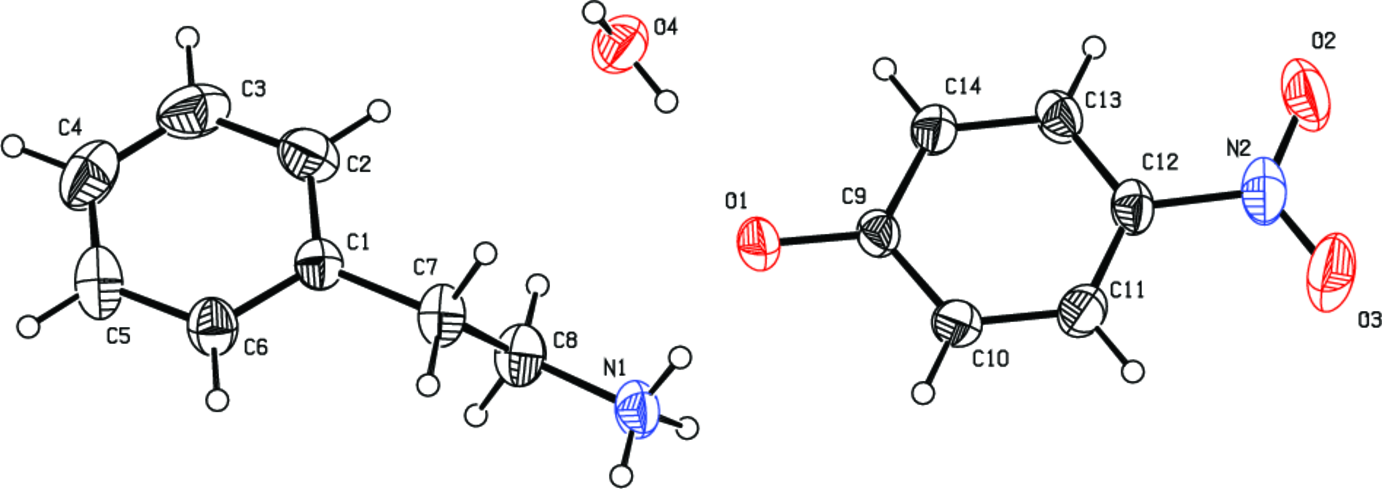

Supplement: Supplementary file 4 [file e-70-o1280-fig1.tif]

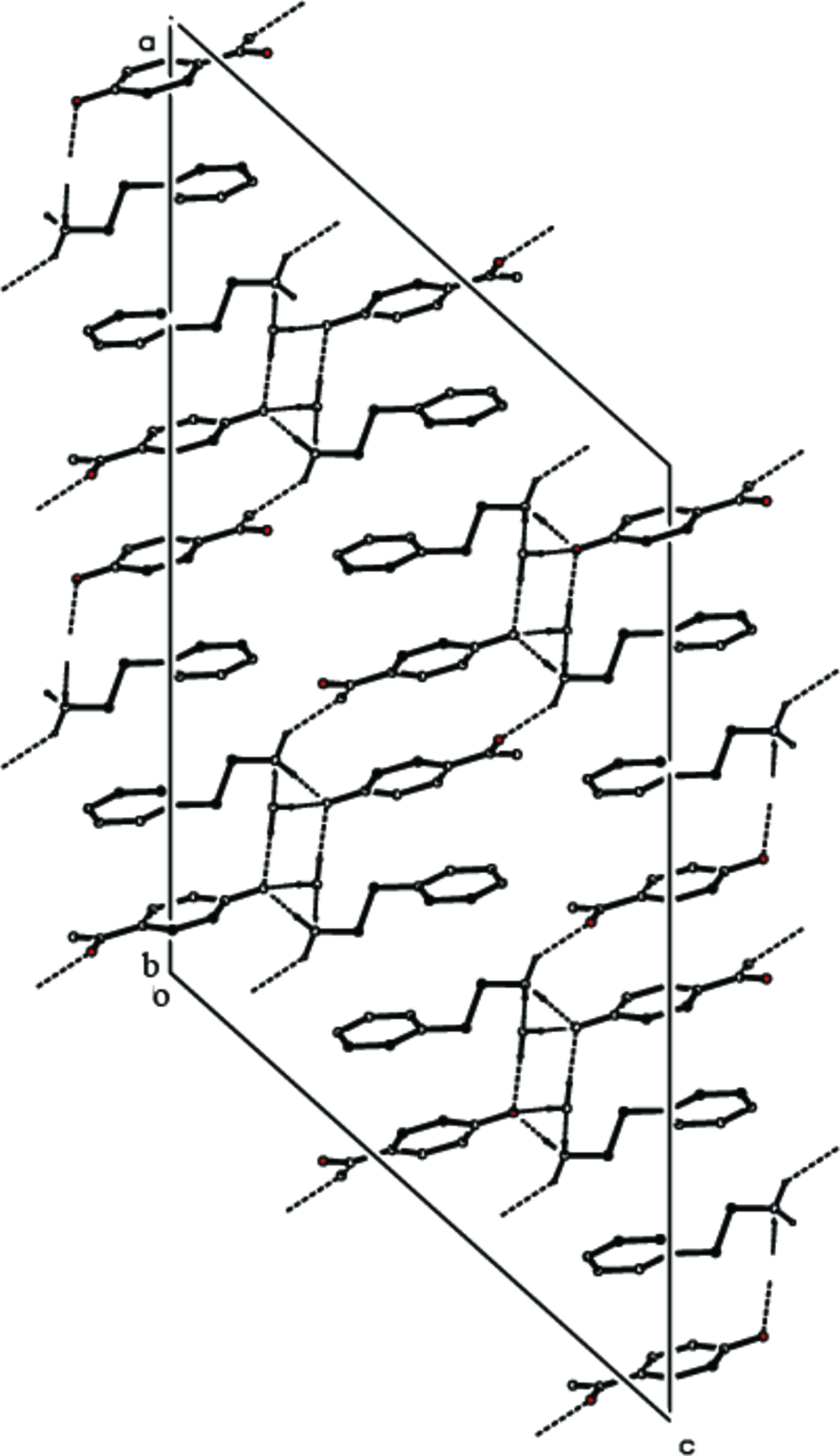

Supplement: Supplementary file 5 [file e-70-o1280-fig2.tif]
